# Effects of urea supplementation on ruminal fermentation characteristics, nutrient intake, digestibility, and performance in sheep: A meta-analysis

**DOI:** 10.14202/vetworld.2022.331-340

**Published:** 2022-02-15

**Authors:** Teguh Wahyono, Mohammad Miftakhus Sholikin, Yusuf Konca, Taketo Obitsu, Sadarman Sadarman, Anuraga Jayanegara

**Affiliations:** 1 National Research and Innovation Agency of Indonesia, Jakarta 10340, Indonesia; 2 Animal Feed and Nutrition Modelling Research Group, Faculty of Animal Science, IPB University, Bogor 16680, Indonesia; 3 Department of Animal Science, Faculty of Agriculture, Erciyes University, Kayseri 38039, Turkey; 4 Department of Animal Science, Graduate School of Biosphere Science, Hiroshima University, 1-4-4 Kagamiyama, Higashi-Hiroshima, Hiroshima 739-8528, Japan; 5 Department of Animal Science, UIN Sultan Syarif Kasim, Pekanbaru 28293, Indonesia; 6 Department of Animal Nutrition and Feed Technology, Faculty of Animal Science, IPB University, Bogor 16680, Indonesia

**Keywords:** meta-analysis, sheep, supplementation, urea

## Abstract

**Background and Aim:**

As a non-protein nitrogen source, urea is a popular, low cost, and easily obtained protein supplement. The objective of the present study was to perform a meta-analysis of the effects of urea supplementation on rumen fermentation and sheep performance.

**Materials and Methods:**

A total of 32 experiments from 21 articles were compiled into a dataset. The levels of dietary urea varied from 0 to 31 g/kg of dry matter (DM). Parameters observed were rumen fermentation product, nutrient intake, nutrient digestibility, and sheep performance. This dataset was analyzed using a mixed model methodology, with urea supplementation levels as fixed effects and the different experiments as random effects.

**Results:**

Increasing levels of urea were associated with increases (p=0.008) in rumen pH, butyrate (C_4_) production, and ammonia (NH_3_–N) concentration. Urea supplementation had minor effects on total volatile fatty acids (p=0.242), total protozoa (p=0.429), and the microbial N supply (p=0.619), but tended to increase methane production (CH_4_; p<0.001). Supplementation of urea increased the intake of dry matter (DM; p=0.004) and crude protein (CP; p=0.001). Digestibility parameters, such as DM digestibility (DMD) and CP digestibility (CPD), also increased (p<0.01) as a result of urea supplementation. Retained N (p=0.042) and N intake (p<0.001) were higher with increasing levels of urea supplementation. In terms of animal performance, supplementation of urea increased average daily gain (ADG; p=0.024), but decreased the hot carcass weight percentage (p=0.017).

**Conclusion:**

This meta-analysis reports the positive effects of urea supplementation on rumen fermentation products (i.e., pH, C_4_, and NH_3_–N), intake (DM, CP, and N), digestibility (DMD and CPD), and ADG in sheep.

## Introduction

In rural development, sheep are one of the most important livestock animals supporting economic activities; therefore, strategies to increase sheep production through feed supplementation must be carefully considered. As a non-protein nitrogen (NPN) source, urea is a reasonable protein replacement due to its low cost, ease of obtainability, and high N density [1]. Furthermore, urea is a popular N source for small and industrial farming because of its lower cost per unit of N compared with other protein sources [2]. Urea is utilized by rumen microbes, which convert it to ammonia and subsequently to microbial protein, thus increasing the supply of protein available to the host [3].

Various studies have generally reported positive effects of urea application as a feed supplement for ruminants [3-6]. Urea supplementation to animals may stimulate nutrient digestibility and improve performance and carcass yield [4]. Urea at 10 g/kg of dry matter (DM) can be used as a substitute for 75% of soybean meal in fattening lambs without decreasing nutrient utilization, rumen fermentation, or animal performance [5]. In addition, the supplementation of urea (10–15 g/kg DM) improves the digestibility of DM, organic matter (OM), and crude protein (CP). In sheep, it increases rumen microbial N, ammonia, and volatile fatty acid (VFA) concentration [6]. Conversely, some studies have reported that urea has no positive effects on sheep [1,2]. For instance, in one study, urea supplementation (3.5 g/kg DM) was shown to have no effect on DM intake (DMI) or ruminal available N [2]. In another study, supplementation of 25 g/kg DM urea in a 50:50 concentrate-to-roughage ratio decreased the DMI and average daily gain (ADG) of sheep [1].

The utilization efficiency of N supplementation may vary according to the different methods and levels of supply [3]. In addition, differences in research design, statistics, animal breed, and other technical conditions can produce variation in experimental results. These challenges can be overcome with the use of meta-analysis, a thorough statistical procedure for analyzing a combined dataset obtained from multiple research experiments [7,8]. Sauvant *et al*. [7] reported that many publications conducted meta-analysis of N supplementation and proved it widely accepted, especially in ruminant nutrition. A recent meta-analysis study conducted by Salami *et al*. [8], who reported that the effects of slow-release urea (SRU) supplementation supported improvements in the live weight gain (LWG) and feed efficiency (FE) in beef cattle.

To the best of our knowledge, there is no published meta-analysis of the effect of urea supplementation in sheep. Although both cattle and sheep are ruminants, sheep can change digestible N into absorbed amino acid–N at a much greater rate than cattle [9]. Furthermore, although N intake is positively correlated with fecal N excretion in all types of ruminants, in sheep, this effect is not statistically significant [10].

Therefore, the present meta-analysis aimed to investigate the influence of urea supplementation on rumen fermentation, nutrient utilization, and production performance in sheep.

## Materials and Methods

### Ethical approval

This is a meta-analysis of the published studies and ethical approval is not required for this study.

### Metadata development

A dataset was compiled from published experiments that reported the influence of urea supplementation on rumen fermentation, nutrient intake, digestibility, and performance in sheep. Literatures were collected from the period 1996 to 2019. The included studies were obtained from the Scopus because it is considered as one of the most comprehensive electronic databases of scientific articles. The articles were identified through searches using “urea,” “supplementation,” and “sheep” as keywords. Inclusion criteria for an article were as follows: (1) The article was published in English; (2) experiments were performed based on conventional urea, not SRU, as described by Sevim and Önol [11]; (3) dietary urea supplementation level was reported; and (4) all parameters were directly measured rather than estimated using predictive equations. After abstract and full-text evaluations, a total of 21 articles (describing a total of 32 experiments) met the inclusion criteria (Table-1) [1-6,12-26]. When a published study reported more than 1 experiment, each individual entity was encoded separately. All experiments were performed directly (*in vivo*) and any *in vitro* experiments using rumen fluid taken from sheep were excluded from the study. The concentration of urea in the present meta-analysis ranged from 0 to 31 g/kg DM, and the experimental periods varied from 15 to 85 days.

**Table 1:** Experiments included in the meta-analysis of the effect of urea supplementation on rumen fermentation and sheep performance.

Study no.	Reference	Method	Basal feed	Breed of sheep	Addition level (g/kg dry matter)
1	[18]	*In vivo*	Eragrostis and lucerne hay-sunflower meal and ground maize (43:57 w/w)	Merino	0 and 10
2	[3]	*In vivo*	Maize stover, ground corn, and cottonseed meal	Merino	0; 7; and 15
3	[1]	*In vivo*	Soybean hull, corn, soybean meal, and wheat bran	Dorper×Thin tailed	0; 5; 15; and 25
4	[20]	*In vivo*	TMR pellet	Poll Dorset Sire×Dohne	0 and 15
5	[2]	*In vivo*	Hard fescue (*Festuca trachyphylla*) straw	Rambouillet×Polypay	0 and 3.5
6	[6]	*In vivo*	Berseem (*Trifolium alexandrinum*) hay-concentrate (60:40 w/w)	Barki	0; 10; and 15
7	[14]	*In vivo*	Barley, corn, soybean meal, and barley straw	Assaf	0; 6; and 9.5
8	[5]	*In vivo*	Corn silage, peanut vine, and corn grain	Hu	0; 10; 20; and 30
9	[22]	*In vivo*	Oat hay	Rambouillet×Kaghani	0; 5; and 10
10	[12]	*In vivo*	Dwarf elephant grass (*Pennisetum purpureum* Schum. Cv. Mott) hay	Polwarth×Texel	0 and 10
11	[23]	*In vivo*	Grass hay (*Cynodon* sp.)	Polwarth×Texel	0 and 10
12	[24]	*In vivo*	Wheat straw, barley grain, wheat grain, wheat bran, and sunflower meal	Merino	0; 6; 12; and 18
13	[16]	*In vivo*	*Acacia saligna* and wheat straw	Merino	0 and 10
14	[15]	*In vivo*	Timothy hay and ground corn	Corriedale×Suffolk	0 and 31
15	[26]	*In vivo*	Para grass and soybean meal	Phan Rang	0 and 18
16	[19]	*In vivo*	Timothy hay and soybean meal	Corriedale×Suffolk	0 and 9
17	[4]	*In vivo*	Buffelgrass	-	5; 8; 11; and 14
18	[13]	*In vitro*	Cotton straw and maize meal	Karakul	0; 10; 20; and 30
19	[17]	*In vivo*	Oat or barley straw	Merino×Romney	0 and 10
20	[25]	*In vivo*	Oaten chaff hay, oats, and lupins	Merino	0 and 20
21	[21]	*In vivo*	Veld hay or Napier hay	Dorper×Merino	0; 10; and 20

The rumen fermentation parameters included in the dataset were pH, total VFA, acetate (C_2_), propionate (C_3_), butyrate (C_4_), isobutyrate (isoC_4_), valerate (C_5_), isovalerate (isoC_5_), ammonia N (NH_3_–N), methane emissions (CH_4_), total protozoa, and microbial N supply. The included nutrient intake and digestibility parameters were DMI, OM intake (OMI), CP intake (CPI), metabolizable energy intake (MEI), non-fiber carbohydrate intake (NFCI), neutral detergent fiber intake (NDFI), acid detergent fiber intake (ADFI), DM digestibility (DMD), OM digestibility (OMD), CP digestibility (CPD), non-fiber carbohydrate digestibility (NFCD), neutral detergent fiber digestibility (NDFD), acid detergent fiber digestibility (ADFD), N intake, and retained N. The assessed sheep performance parameters were ADG, FE, hot carcass weight (HCW), and cold carcass weight (CCW).

### Preferred reporting items for systematic reviews and meta-analyses (PRISMA) guideline

**Figure-1:**
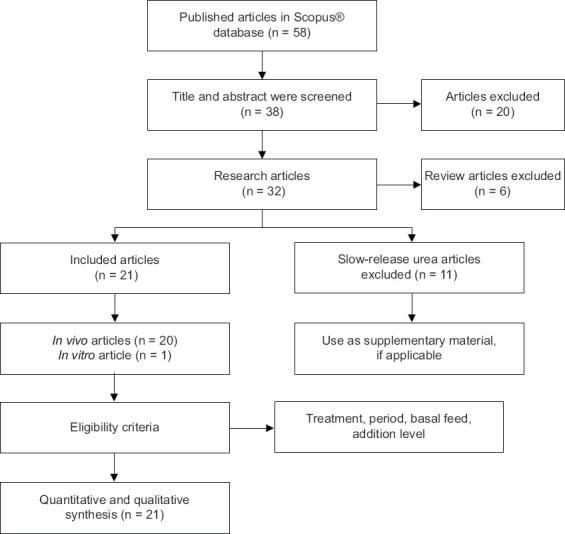
Diagram of literature search.

This study used the protocol described by PRISMA guidelines [27]. We selected and extracted the data according to the PRISMA protocols (Figure-1).

### Statistical analysis

A meta-analysis was performed using a statistical analysis based on a mixed model methodology [28,29]. Accordingly, different studies were treated as random effects, whereas the levels of urea supplementation in sheep diets were treated as fixed effects. The mathematical models used were as follows:


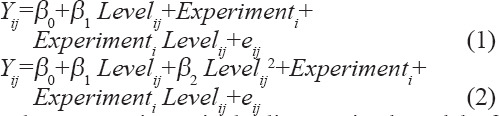


Where, Equation 1 is the linear mixed model of the 1^st^ order (linear model), and Equation 2 is the linear mixed model of the 2^nd^ order (quadratic model). Model statistics used were p-value and root mean square error (RMSE). The result was significant if the p-value was less than or equal to 0.05, and if the p-value ranged from 0.05 to 0.1, there was a tendency to be significant. The model implemented was the quadratic (2^nd^ order) version, and this was then assessed to its linear model (1^st^ order) when the quadratic term was non-significant. All statistical analyses were performed in R software version 3.6.3 equipped with an “nlme” library [30].

## Results

### Effect of urea supplementation on rumen fermentation in sheep

The effect of urea supplementation on the rumen fermentation parameters is outlined in Table-2. Increasing urea levels were associated with an increase in rumen pH (p=0.008). The addition of urea did not change the total VFA production. With regard to VFA composition, the proportions of C_4_ and iso C_4_ increased (p=0.033) and decreased (p=0.006), respectively, as a result of urea supplementation. Urea supplementation did not change the proportions of C_2_, C_3_, C_5_, iso C_5_, or C_2_:C_3_. An increase in rumen NH_3_–N (p=0.012) and enteric CH_4_ (p=0.0006) was associated with increasing levels of urea supplementation. Urea supplementation had a minor effect on the total protozoa population (p=0.429) and microbial N supply (p=0.619).

**Table 2:** Influence of urea supplementation (g/kg dry matter intake) on rumen fermentation of sheep.

Response parameter	Unit	n	Intercept	SE intercept	Slope	SE slope	p-value	RMSE
pH	-	40	6.49	0.086	0.007	0.002	0.008	1.191
Total VFA	mmol/L	26	9.46	0.453	0.019	0.016	0.242	1.117
C_2_	mol/100 mol	24	7.59	0.297	–0.006	0.008	0.469	1.232
C_3_	mol/100 mol	24	4.76	0.212	–0.011	0.009	0.269	1.331
C_4_	mol/100 mol	24	3.21	0.252	0.010	0.004	0.033	1.002
isoC_4_	mol/100 mol	15	1.12	0.106	–0.011	0.003	0.006	0.821
C_5_	mol/100 mol	19	1.34	0.104	0.002	0.002	0.327	1.023
isoC_5_	mol/100 mol	15	1.23	0.141	0.002	0.002	0.348	0.911
C_2_/C_3_	-	24	1.79	0.080	0.003	0.003	0.309	1.038
NH_3_-N	mmol/L	34	2.88	0.355	0.071	0.012	0.012	1.584
CH_4_	g/kg BW^0.75^	6	1.36	0.028	0.015	0.001	<0.001	0.550
Total protozoa	log/mL	6	3.17	0.464	-0.018	0.020	0.429	0.776
Microbial N supply	g/d	6	1.99	0.383	0.021	0.036	0.619	1.191

VFA=Volatile fatty acid, C_2_=Acetate, C_3_=Propionate, C_4_=Butyrate, C_5_=Valerate, NH_3_=Ammonia, CH_4_=Methane, SE=Standard error, RMSE=Root mean square error

### Effect of urea supplementation on nutrient intake and digestibility in sheep

Urea supplementation increased the intake of DM (p=0.004), ME (p=0.013), and CP (p=0.001), but did not influence OMI, NFCI, NDFI, or ADFI (Table-3). Digestibility parameters, such as DMD and CPD, were increased by urea supplementation (p<0.01), and NFCD was higher with increasing levels of urea supplementation (p=0.028). Supplementation of urea did not change the OMD, NDFD, or ADFD. There was an increase in N intake (p<0.001) and retained N (p=0.042) with increasing urea levels.

**Table 3:** Influence of urea supplementation (g/kg DMI) on nutrient intake and digestibility of sheep.

Response parameter	Unit	n	Intercept	SE intercept	Slope	SE slope	p-value	RMSE
DMI	g/kg BW^0.75^	58	7.98	0.295	0.023	0.008	0.004	1.178
OMI	g/kg BW^0.75^	25	8.11	0.359	0.007	0.008	0.419	0.918
CPI	g/kg BW^0.75^	19	2.77	0.431	0.034	0.008	0.001	0.799
MEI	g/kg BW^0.75^	6	15.82	2.242	0.495	0.056	0.013	0.569
NFCI	g/kg BW^0.75^	7	4.15	0.630	0.004	0.006	0.581	0.677
NDFI	g/kg BW^0.75^	25	5.95	0.204	-0.003	0.008	0.744	1.208
ADFI	g/kg BW^0.75^	17	4.41	0.079	0.001	0.004	0.919	0.996
DMD	g/kg	47	23.34	0.470	0.024	0.014	0.096	1.297
OMD	g/kg	36	24.09	0.487	0.026	0.017	0.130	1.241
CPD	g/kg	19	25.54	0.807	0.086	0.027	0.009	1.160
NFCD	g/kg	3	27.82	0.068	0.080	0.004	0.028	0.496
NDFD	g/kg	30	21.88	0.785	-0.005	0.024	0.854	1.117
ADFD	g/kg	21	18.91	1.238	0.009	0.033	0.799	0.496
N intake	g/kg BW^0.75^	29	1.28	0.061	0.016	0.004	<0.001	1.117
Retained N	g/kg N intake	20	14.32	1.317	0.256	0.109	0.042	1.152

DMI=Dry matter intake, OMI=Organic matter intake, CPI=Crude protein intake, MEI=Metabolizable energy intake, NFCI=Non-fiber carbohydrate intake, NDFI=Neutral detergent fiber intake, ADFI=Acid detergent fiber intake; DMD=Dry matter digestibility, OMD=Organic matter digestibility, CPD=Crude protein digestibility, NFCD=Non-fiber carbohydrate digestibility, NDFD=Neutral detergent fiber digestibility, ADFD=Acid detergent fiber digestibility, SE=Standard error, RMSE: Root mean square error

### Effect of urea supplementation on sheep performance

Supplementation of urea increased ADG (p=0.024), but decreased HCW percentage (p=0.017; Table-4). Conversely, FE, CCW, and dressing percentage were not influenced by urea supplementation.

**Table 4:** Influence of urea supplementation (g/kg DMI) on sheep performance.

Response parameter	Unit	n	Intercept	SE intercept	Slope	SE slope	p-value	RMSE
ADG	g/kg BW^0.75^	47	3.39	0.254	0.023	0.009	0.024	1.108
FE	35	4.16	0.102	0.003	0.003	0.382	1.226
HCW	%	8	5.68	1.063	–0.006	0.002	0.017	0.768
CCW	%	27	5.04	0.152	–0.001	0.002	0.611	1.080
Dressing	%	23	7.07	0.023	–0.002	0.002	0.336	1.207

ADG=Average daily gain, FE=Feed efficiency, HCW=Hot carcass weight, CCW=Cold carcass weight, SE=Standard error, RMSE=Root mean square error

## Discussion

The aim of the present study was to investigate the effect of urea supplementation on rumen fermentation, intake, nutrient digestibility, and production performance in sheep. According to Khattab *et al*. [6], rumen fermentation products are represented in terms of pH, NH_3_–N concentration, and total VFA composition; this is especially true for NH_3_–N, which is an important nitrogen source for microbial protein synthesis and growth in the rumen [5]. Productive performance (ADG, HCW, and CCW) is an important indicator of sheep production [4]. Nutrient utilization can also be represented in the form of intake and nutrient digestibility parameters [2].

### Effect of urea supplementation on rumen fermentation in sheep

Ruminal pH is the first indicator representing the fermentation characteristics in the rumen. Fermentation and chemical rate differences between supplemental feed sources are reflected by pH curves [12]. The present meta-analysis study revealed an increase in the rumen pH of sheep following urea supplementation. Supplementation of urea at 3.5-31 g/kg increased the pH value from 0.04 to 0.36 of the initial value. The pH values remained within a normal range of 5.5-7.0 [31,32]. Aschenbach *et al*. [32] reported that NPN fermentation in the rumen may release excess NH_3_–N and thus increase pH. Furthermore, rumen bacteria convert urea into increased ruminal NH_3_–N, which is a potent buffer. Ammonia accumulation from urea degradation may increase ruminal pH [13]. The rate of ureagenesis determines the disposal of bicarbonate and affects the maintenance of pH homeostasis [14].

In the present meta-analysis study, increasing pH values were concomitant with increases in NH_3_–N concentration after urea supplementation. It is widely known that urea provides a source of N for microbial protein synthesis. Other studies have reported that the rumen concentration of NH_3_–N increases linearly with increasing NPN supplementation levels [5,15]. The level of NH_3_–N in the ruminal fluid is important since it greatly affects rumen microbial growth [6]. As a rumen-degradable protein source, urea can increase microbial CP synthesis production and thus increase the metabolizable protein supply to host animals [13].

Moreover, more than 50% of dietary N is passed directly through the NH_3_–N pool in sheep [33]. Theoretically, urea supplementation increases the availability of N sources from diets with varying protein contents. Nonetheless, it is interesting to note that an increase in NH_3_–N levels due to urea supplementation occurred in both low-quality [3,16,17] and high-quality diets [5,6,15,18,19]; this may be connected to the theory that the length of time during which ruminal microbes have access to N source substrate is reduced [2], thereby potentially increasing protein synthesis by bacteria [6] and protozoa [13]. Ruminants that consume low-quality forage efficiently use urea as a source of N supplementation [3]; however, urea supplementation should be balanced with the intake of soluble carbohydrates. Kozloski *et al*. [23] reported that sheep supplemented with urea alone had an increased N intake; however, most of this additional N was excreted in the urine. The minimum NH_3_–N requirement for microbial growth and activity depends on carbohydrate availability. As such, dietary manipulation should be conducted to obtain the optimal ruminal energy supply and provide the appropriate amount of available N [34].

Ruminal NH_3_–N is an equilibrium between dietary N degradation, microbial protein synthesis utilization, and N absorption. Greater NH_3_–N production from dietary sources is not necessarily reflected in a higher ruminal NH_3_–N concentration [14]. The current meta-analysis shows urea supplementation had a minor effect on the microbial N supply and total protozoa population. In agreement with the present findings, Zhao *et al*. [3] reported that urea supplementation does not affect microbial N or escaped N from feed. This effect is probably due to the regulation of bacterial equilibrium in the rumen ecosystem. The high-fiber content in some basal diets does not cause dramatic changes in microbial growth because it is slowly ingested [12]. For example, under dry pasture conditions, energy source supplements are more necessary than nitrogen supplements to accelerate microbial protein synthesis in the rumen for urea [35]. The lack of change in total protozoa is likely due to the interrelationships between protozoa and ruminal bacteria in the N-cycle. Protozoa engulf large numbers of ruminal bacteria and compete with ruminal bacteria for nutrients [36]. The total protozoa population was not affected by dietary urea supplementation [20]. Although not significant, the microbial N supply still showed a positive trend. A lack of significant differences in the microbial N supply and total protozoa population indicates that, in urea-supplemented diets, the adaptation factor related to microbial ecosystem changes must be monitored. Since there was no change in the protozoa population nor the microbial N supply, the molar proportion of total VFAs did not change. In agreement with the present study, Xu *et al*. [5] noted that similar concentrations of acetate, propionate, or total VFAs could be due to a similar proportion of carbohydrates in the diets. The relative concentration of total VFAs is often assumed to represent the carbohydrate fermentation rate and microbial conditions in the rumen [31]. Despite the effect on total VFAs, acetate and propionate were not affected by urea supplementation, and the butyrate proportion experienced a relatively positive effect. As such, an increase in butyrate formation through urea supplementation may explain the role of butyrates in enhancing N degradation in the rumen [36]. Butyrate/butyric acid increases the total urea synthesis and is degraded in the rumen and stimulates the transfer of urea to the gastrointestinal tract [37]. The present meta-analysis study reveals a positive association between urea degradation and the butyrate proportion.

The increased tendency for higher CH_4_ production at higher levels of urea supplementation appears to be related to the increased utilized N availability, which is used by methanogenic archaea. A previous study revealed that CH_4_ production was positively correlated with the microbial population and NH_3_–N concentration [38]. Conversely, Bharanidharan *et al*. [39] revealed that rumen metabolites and NH_3_–N concentration correlate negatively (p<0.001) with methane production. Furthermore, different methods of feeding (total mixed rations [TMRs] or concentrate and roughage fed separately) lead to different implications with regard to the relationship between N utilization and CH_4_ production. Another explanation for this higher CH_4_ production in the presence of urea supplementation may be due to the interrelationship between butyrate and the CH_4_ pathway. A simultaneous increase in butyrate (producing H_2_) and decrease in isobutyrate affects the regulation of H_2_ in the production of CH_4_. In agreement with the present findings, a study by Granja-Salcedo *et al*. [40] reported that long-term encapsulated urea supplementation increases the butyrate proportion and positively correlates with *Archaea Euryarchaeota* (*Methanobacterium*, *Methanobrevibacter*, and *Methanomassiliicoccus*). In addition, Dong *et al*. [41] also observed that *Methanobrevibacter* has a positive (p<0.05) relationship with butyrate production.

Furthermore, this explanation reflects the correlation between methanogenesis function and ruminal fermentation variables. Enteric CH_4_ emission represents a loss of energy from ruminants, which could be potentially (at least partially) utilized for production and reproduction [42]. Partitioning of the fermented OM between ruminal microbes, total VFA, and CH_4_ depends on: (1) The availability of substrates and pathways of cellular material synthesis; (2) the adenosine triphosphate requirements of the microbes; and (3) the turnover of microbial cells within rumen ecosystems [43].

### Effect of urea supplementation on nutrient intake and digestibility in sheep

DMI, CPI, and MEI increased with dietary urea supplementation. These results may be attributed to the increase of ruminally available N after the NH_3_–N concentration increased. The optimal level of available N can increase microbial growth and apparently increase dietary intake. The addition of urea can increase DMI and CPI due to an improvement in microbial fermentation when the NH_3_–N concentration increases in the rumen [5,44], thus improving availability in the intestines [45]. As discussed above, urea supplementation can increase the pH in the rumen and have a positive effect on the absorption of rumen NH_3_–N into the blood [5]. Variable results have been reported by other authors; For instance, Wang *et al*. [1] reported that DMI is reduced in Dorper crossbreed sheep after urea supplementation treatment of up to 25 g/kg DM. In addition, Zhao *et al*. [3] suggested that the response to urea supplementation may be lower if the diet contains high CP levels and results in higher NDF intake.

Conversely, Manyuchi *et al*. [21], McGuire *et al*. [2], and Xu *et al*. [5] stated that DMI and CPI are higher in sheep who receive urea supplementation than in non-supplemented animals. Different results were shown by Kozloski *et al*. [12] and Currier *et al*. [46], who reported that supplemental urea to ruminants consuming low-quality roughage does not affect an animal’s DMI. Although there was no change in DMI, we assume a change in the intake of other nutrient components. There are several explanations for these differing nutrient intake results related to urea supplementation in sheep, including: (1) Differences in CP concentration and the quality of the original basal diets [1]; (2) differences in the chemical and/or physical characteristics of the forage/feedstuff [15,47]; and (3) differences in the slow-fast degradable carbohydrate and/or forage-concentrate composition of the basal diets [3,44].

Accordingly, increased feed intake in response to urea supplementation may be attributed to the mechanism of N and C synchronization utilization in the rumen, which can increase the rate of microbial growth [5]. Urea supplementation may not clearly influence protein synthesis in animal feed isoenergetic concentrate diets, even though the fermentation characteristics of the rumen are changed [15]. Notably, urea supplementation can increase resilience to parasitism, thereby improving feed intake and enhancing resistance mechanisms against *Haemonchus contortus* and *Trichostrongylus colubriformis* worms in sheep on low-quality diets [45]. Furthermore, it is reasonable to conclude that high DMI resulted from enhanced host immunological responses arising from an improved nutritional status after urea supplementation. Urea has also been reported to improve N intake in diets rich in tannin-containing tree leaves, by providing extra N sources for ruminal microbes [22]. Moreover, tannin activity can be neutralized [48] and reduced [49] by urea supplementation.

Based on this meta-analysis, it is apparent that urea supplementation influences DMD, CPD, and NFCD. The increase in DMI and CPI induces positive feedback on nutrient digestibility in the digestive tract of sheep. Urea supplementation benefits the function of ruminal microbes and thus improves ruminant production [19]. Moreover, urea supplementation has been shown to improve total tract digestibility in sheep consuming low-quality forage [2]. Another explanation is that urea can increase resilience to parasitism because of improved nutritional status. Adding 3% urea to basal diets can increase feed digestibility by 10-15% [45]. Although urea improves digestibility in various types of diets, McGuire *et al*. [2] and Dixon *et al*. [17] reported that urea supplementation is more optimally applied to diets based on low-quality forage; increasing the availability of NPN sources would help to address the lack of CP content derived from low-quality forage. Small quantities of essential nutrients, including urea, can perform acceptably with low digestibility forage [17,43,46]. Apparently, from the perspective of digestibility, low urea levels (<3 g/kg DM) are optimally used in diets based on low-quality forage. In contrast, high urea levels (up to 30 g/kg DM) are effectively applied to high concentrate/TMR-based diets. There is a need for further evaluations of this hypothesis. Urea is also likely useful when given with low-protein supplements that do not satisfy N microbial requirements from degraded fractions [50]. In a previous study, feed intake did not always positively affect digestibility: Fecal DM excretion increased as nutrient intake increased, indicating a greater passage of digesta after N supplementation [47]. Zhao *et al*. [3] found that direct urea spray added to maize stover (up to 39.5% of a dietary level) reduced the digestibility of nutrients. High urea levels supplemented in diets may cause N loss during micturition and decrease N utilization efficiency [1]. However, this explanation differs from the current results of our meta-analysis; this difference could be due to variations in basal diet characteristics.

In addition, CPD is positively correlated with dietary CP level when metabolic fecal N is constant [5]. Apparent total tract N digestibility for urea-supplemented treatments is approximately 110% higher than for unsupplemented treatments [46]. Accordingly, CPD increases following the increase in N availability resulting from urea supplementation. As such, CPD may have a positive relationship with N intake and retained N. The greater digestibility of N supplements indicates that the proportion of N intake increased in line with the decrease in metabolic fecal N [2]. Nonetheless, the mechanism by which N retention is higher under urea supplementation than unsupplemented treatment is unclear.

Moreover, urea supplementation may increase feed degradability, thus increasing sugar availability for improved nutritional status [23]. Furthermore, greater N retention is caused by higher MEI and higher microbial protein entering the small intestine in sheep. The characteristic rapid degradation of urea will increase N utilization from the fermentation process in the rumen. Increases in total ruminal degradable N were most likely due to the decomposition of supplemented urea in the rumen [3]. We must, therefore, consider that N retention and digestibility were positively affected by the characteristics of the base feed [47] and the proportion of N to non-structural carbohydrates in the diets [11].

### Effect of urea supplementation on sheep performance

Urea supplementation had a positive effect on ADG. Supplemental urea also numerically (p=0.382) increased FE for a better feed cost to gain ratio. Based on the present study, it is clear that urea can increase DMI, CPI, DMD, and CPD. The increases in intake and digestibility are generally known to positively affect body weight gain, except for carcass quality. This increase in fattening performance is related to the increase in CPD, due to rumen microbes having a quickly available N source from urea [24]. Accordingly, improving microbial growth in the rumen may increase daily body weight gain. In addition, animal growth performance is likely enhanced by supplementing diets with urea at levels beyond that necessary for maximal rumen microbial growth [51]. As explained above, urea supplementation also gives rise to immunological responses in sheep [45]. When sheep are raised under good health conditions, their ADG primarily depends on their DMI and nutrient digestibility [5]. Urea supplementation also promotes increased feed intake to help sheep maintain their body condition, thus improving their final body weight gain [25]. In contrast, Wang *et al*. [1] stated that urea supplementation at 25 g/kg DM may reduce the growth performance of Dorper sheep fed a diet with a 50:50 concentrate to forage ratio. We must consider that the urea degradation rate could be more rapid than the NH_3_–N utilization rate by rumen microbes, leading to ruminal accumulation and absorption. Therefore, it is necessary to determine the optimal level of urea supplementation for the specific diet (characteristics and/or ratio) fed to the sheep breed of interest [5]. With a different form of urea (SRU), NPN supplementation exhibits consistent improvement in the LWG and FE of beef cattle [10].

Urea supplementation had a minor effect on CCW and dressing percentage; however, the HCW parameter tended to decrease. This decrease in carcass value appears to be associated with increased non-carcass values caused by urea supplementation. The fat percentage of the kidneys, pelvic area, and heart increase linearly with increasing urea supplementation [52]. Unfortunately, non-carcass proportion was not widely reported in the literature used for this meta-analysis and thus could not be integrated into the dataset and analysis; thus, this explanation remains unclear. In agreement with the present meta-analysis findings, a study by Cosby and Stanton [53] reported that although the feed cost of gain is lowest for a urea-supplemented diet, natural protein supplemented in basal diets is still competitive and creates greater carcass weight compared to urea supplementation. To the best of our knowledge, the present study is the first to apply a meta-analysis to objectively review the effect of urea supplementation on the performance of sheep. There is an important role for well-designed research experiments to provide efficacy results that can support farmers and industry users in making the most profitable and efficient sheep farm management decisions.

## Conclusion

This meta-analysis revealed that urea, as a low-cost N supplement, is a reasonable choice for improving rumen fermentation, nutrient intake, digestibility, and sheep performance. Urea supplementation consistently improves NH_3_–N production, CPI, CPD, N intake, and ADG in sheep; however, urea supplementation also tends to increase enteric CH_4_ emissions and decreases carcass performance. This study is limited to the findings available in the Scopus database so, further studies should be conducted to involve the various findings from various databases.

## Authors’ Contributions

TW: Investigation and writing of the manuscript. MMS: Methodology and software experiments, formal analysis, and visualization. YK and TO: Conceptualization and validation. SS: Revised the manuscript. AJ: Conceptualization, data curation, writing of the manuscript, supervision, and validation. All authors read and approved the final manuscript.
